# Outbreaks and incidence of vector-borne diseases in Colombia (2007-2024): Impact of climate change and deforestation

**DOI:** 10.7705/biomedica.7897

**Published:** 2025-11-27

**Authors:** Mario J. Olivera, Julián Felipe Porras-Villamil, Màrius Vicent Fuentes

**Affiliations:** 1 Grupo de Parasitología, Dirección de Investigación en Salud Pública, Instituto Nacional de Salud, Bogotá, D. C., Colombia Instituto Nacional de Salud Instituto Nacional de Salud Bogotá, D. C. Colombia; 2 Grupo de Investigación Parásitos y Salud, Facultat de Farmàcia i Ciències de l’Alimentació, Universitat de València, València, España Universitat de València Universitat de València València Spain; 3 Facultad de Ciencias de la Salud, Universidad de la Salle, Bogotá, D. C., Colombia Universidad de la Salle Universidad de la Salle Bogotá, D. C. Colombia

**Keywords:** Vector borne diseases, climate change, conservation of natural resources, epidemiologic studies, Colombia, enfermedades transmitidas por vectores, cambio climático, conservación de los recursos naturales, estudios epidemiológicos, Colombia

## Abstract

**Introduction.:**

Vector-borne diseases pose a public health challenge in Colombia, influenced by climatic and environmental factors. El Niño and deforestation can alter vector habitats, affecting the incidence of dengue, Zika, chikungunya, malaria, cutaneous leishmaniasis, and yellow fever. This study analyzes the relationship between these variables and vector-borne diseases incidence in Colombia (2007-2024).

**Materials and methods.:**

An ecological study was conducted using incidence and outbreak data for six vector-borne diseases, linked to climate information, El Niño, and deforestation. Regression models and random forests were applied to assess associations.

**Results.:**

Between 2007 and 2024, 3,283,259 cases of vector-borne diseases were reported in Colombia. Of these, 49.9% (1,639,120) were dengue and 39.8% (1,307,351) malaria, accounting for 89.7% of total cases. El Niño was associated with increased incidence of dengue (β = 213.24; 95% CI: 86.05-338.43), chikungunya (β = 26.41; 95% CI: 17.54-70.36), and Zika (β = 14.12; 95% CI: 10.06-89.30). Maximum temperature showed a positive relationship with dengue (β = 5.74; 95% CI: 2.15-13.63) and malaria (β = 17.28; 95% CI: 3.81-30.75). Deforestation was associated with malaria (β = 12.35; 95% CI: 4.6220.08) and cutaneous leishmaniasis (β = 8.67; 95% CI: 2.21-15.13). Mean precipitation had negative associations with chikungunya and leishmaniasis.

**Conclusions:**

Climate change and deforestation impact the epidemiology of vector-borne diseases in Colombia. Integrated public health and environmental conservation strategies are needed to mitigate their effects.

## Resumen

**Introducción.:**

Las enfermedades transmitidas por vectores representan un desafío para la salud pública en Colombia, influenciadas por factores climáticos y ambientales. El Niño y la deforestación pueden alterar los hábitats de los vectores, afectando la incidencia de dengue, fiebre de chikunguña, enfermedad por el virus del Zika, malaria, leishmaniasis cutánea y fiebre amarilla.

**Objetivo.:**

Analizar la relación entre estas variables y la incidencia de las enfermedades transmitidas por vectores en Colombia (2007-2024).

**Materiales y métodos.:**

Se realizó un estudio ecológico utilizando los datos de incidencia y brotes de seis enfermedades transmitidas por vectores, vinculados con información climática, El Niño y deforestación. Se aplicaron modelos de regresión y bosques aleatorios para evaluar asociaciones.

**Resultados.:**

Entre el 2007 y el 2024, se registraron en Colombia 3'283.259 casos de enfermedades transmitidas por vectores. De estos, el 49,9 % (1’639.120) correspondieron a dengue y el 39,8 % (1’307.351) a malaria, representando el 89,7 % del total. El Niño se asoció con un aumento en la incidencia de dengue (β = 213,24; IC 95%: 86.05-338.43), fiebre de chikunguña (β = 26,41; IC 95%: 17,54-70,36) y enfermedad por el virus del Zika (β = 14,12; IC 95%: 10,06-89,30). La temperatura máxima mostró una relación positiva con el dengue (β = 5,74; IC 95%: 2,15-13,63) y la malaria (β = 17,28; IC 95%: 3,81-30,75). La deforestación se asoció con malaria (β = 12,35; IC 95%: 4,62-20,08) y leishmaniasis (β = 8,67; IC 95%: 2,21-15,13). La precipitación media tuvo asociaciones negativas con fiebre de chikunguña y leishmaniasis.

**Conclusiones.:**

El cambio climático y la deforestación impactan la epidemiología de las enfermedades transmitidas por vectores en Colombia. Se requieren estrategias integradas de salud pública y conservación ambiental para mitigar sus efectos.

Vector-borne diseases pose a persistent challenge to public health in Colombia and other tropical regions ^1^. Diseases such as dengue, malaria, yellow fever, leishmaniasis, chikungunya, and Zika have shown variable incidence patterns in response to environmental and climatic factors ^2^^,^^3^. Among these factors, climate change and deforestation have been identified as key determinants influencing vector dynamics and, consequently, the spread of these diseases ^2^^,^^3^.

The El Niño-Southern Oscillation (ENSO) is a recurring climate phenomenon that includes both El Niño and La Niña phases, which affect global temperature and precipitation patterns through complex oceanatmosphere interactions ^4^. In Colombia, the El Niño phase has been particularly associated with hotter and drier conditions that favor vector survival and increase the transmission potential of diseases such as dengue, Zika, and chikungunya ^5^. Although La Niña may influence disease dynamics in different ways, this study focused on El Niño given its stronger and more documented association with arboviral outbreaks in the region.

It is also important to note that while these diseases are endemic in Colombia and maintain an expected baseline incidence, certain environmental conditions -such as those induced by El Niño- can amplify transmission, leading to epidemic outbreaks. Similarly, deforestation and other land-use changes create new habitats for vectors, affecting human populations’ exposure to these pathogens and modifying transmission patterns ^6^.

Vector-borne diseases remain one of the leading causes of morbidity in Colombia. According to the latest epidemiological data, dengue is the most prevalent arboviruses, with recurrent epidemic cycles and a significant burden on the healthcare system ^7^^,^^8^. Malaria, though more geographically concentrated in endemic regions, continues to pose a challenge in rural and marginalized communities ^9^^,^^10^. Meanwhile, emerging diseases such as chikungunya and Zika have caused major outbreaks following their introduction into the country, whereas cutaneous leishmaniasis and yellow fever maintain a stable incidence but present a risk of re-emergence in jungle areas ^11^^,^^12^.

While previous studies have explored the relationship between these diseases and environmental factors, most have focused on specific outbreaks or individual diseases ^13^. However, a comprehensive approach is needed to assess both outbreaks and incidence rates over time across multiple vector- borne diseases relevant to public health. Combining statistical models with spatial analysis might provide a deeper understanding of epidemiological patterns and their relationship with climate variability and environmental transformation ^14^.

The objective of this study was to evaluate the impact of ENSO and deforestation on the incidence and outbreaks of six vector-borne diseases (dengue, malaria, yellow fever, leishmaniasis, chikungunya, and Zika) in Colombia from 2007 to 2024. Using robust analytical methodologies, this research aimed to provide evidence on how these factors have shaped the burden of vector-borne diseases in the country, offering key insights for epidemiological surveillance and public health decision-making to design more effective prevention and control strategies.

## Materials and methods

### Study design

An ecological study was conducted based on incidence and outbreak data of vector-borne diseases in Colombia from 2007 to 2024. The six previously mentioned vector-borne diseases (dengue, yellow fever, malaria, cutaneous leishmaniasis, chikungunya, and Zika) were analyzed assessing the relationship between disease events and environmental factors such as ENSO, temperature, precipitation, and deforestation were evaluated.

### Data sources

Vector-borne diseases incidence and outbreak data were obtained from official records of the *Sistema Nacional de Vigilancia en Salud Pública*, SIVIGILA (https://www.ins.gov.co/Paginas/Inicio.aspx). Climatic variables related to temperature (mean, minimum, and maximum) and precipitation (minimum, mean, and maximum) were collected from national and international meteorological databases, including the *Instituto de Hidrología, Meteorología y Estudios Ambientales*, IDEAM (https://www.ideam.gov.co/) and the National Oceanic and Atmospheric Administration (NOAA) (https://www.noaa.gov/). These variables were processed with departmental spatial resolution and monthly temporal resolution to align them with epidemiological data.

Deforestation data were sourced from the Global Forest Watch platform and IDEAM, with spatial resolution at the departmental level and annual temporal resolution. All data were georeferenced and integrated into a unified analytical framework, allowing spatial and temporal analyses to assess the relationship between environmental factors and vector-borne diseases incidence.

### Data analysis

Data was organized and tabulated in a structured database using Microsoft Excel (Microsoft, Redmond, WA, USA), and statistical analyses were performed using R software. Map visualizations were created using QGIS. Continuous variables included the annual incidence of each vector- borne disease, temperature (mean, minimum, and maximum), precipitation (minimum, mean, and maximum), and deforested area. Categorical variables, such as the presence or absence of the El Niño phenomenon and the occurrence of epidemic outbreaks, were presented as frequencies and proportions. Data distribution was assessed, and annualized incidence rates were calculated by department and disease.

Annualized incidence rates were obtained by dividing the number of new cases of each vector-borne disease each year by the at-risk population during the same period, multiplying the result by 100,000 inhabitants. For spatial analysis, incidence rates were estimated at the departmental level using official population projections.

ENSO was operationalized as a binary variable (presence/absence) for each year based on publicly available climatological classifications. Its influence was examined both descriptively and analytically by comparing incidence rates in years with and without ENSO events.

Poisson regression models with a population offset were used to assess the association between vector-borne diseases incidence and environmental variables. Predictors included ENSO, temperature (mean, minimum, and maximum), precipitation (minimum, mean, and maximum), and deforestation. While ENSO is known to be influenced by broader socioeconomic and ecological dynamics, this study focused on its localized climatic expression and statistical association with disease incidence in Colombia.

To evaluate the influence of climate change and deforestation on outbreak incidence, machine learning models using random forests were implemented to determine the relative importance of each predictor variable. Additionally, incidence rates in years with and without ENSO events were compared using parametric and non-parametric statistical tests, complemented by graphical analyses to identify trends and potential associations.

Mucosal and visceral forms of leishmaniasis were excluded, focusing on those with the greatest epidemiological impact in the region. The significance level of p < 0.05 was considered statistically significant for all analyses.

## Results

### Burden of vector-borne diseases in Colombia

Between 2007 and 2024, a total of 3,283,259 vector-borne disease cases were reported in the country. Of these, 49.9% (1,639,120) corresponded to dengue and 39.8% (1,307,351) to malaria, collectively accounting for 89.7% of all reported cases. Cutaneous leishmaniasis represented 4.6% of cases, with a total of 151,038 reports during the study period. Since the introduction of chikungunya in 2014, a total of 78,966 cases were recorded by the end of 2024. Similarly, following the emergence of the Zika virus in 2015, 106,738 cases were reported (table 1). While the overall incidence of Zika and chikungunya has declined after initial epidemic waves, their peak years overlapped with El Niño events, which allowed for statistically significant associations in our models.

**Table 1:** Total cases and outbreak frequency of vector-borne diseases in Colombia (2007-2024)

Disease	Total cases	Percentage of total cases	Years with outbreaks	Percentage of years with outbreaks
Dengue	1,639,120	49.9	8	30.8
Malaria	1,307,351	39.8	5	19.2
Cutaneous leishmaniasis	151,038	4.6	4	15.4
Zika	106,738	3.3	2	7.7
Chikungunya	78,966	2.4	3	11.5
Yellow fever	46	<0.01	4	15.4
Total	3,283,259	100	26	100

### Trends in the incidence of vector-borne diseases in Colombia and their relationship with environmental factors

**Figure 1:**
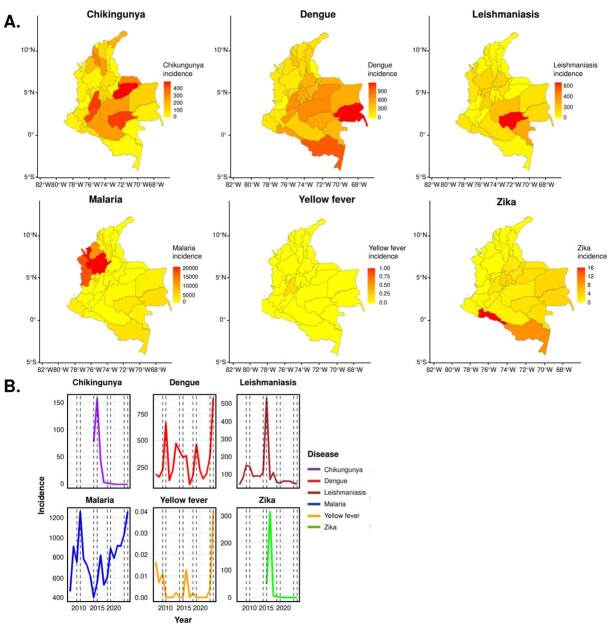
Spatial and temporal patterns of vector-borne disease incidence in Colombia (2007-2023). **A.** Geographic distribution of incidence rates by department. **B.** National incidence trends over time.

The analysis of the influence of climatic and environmental factors on incidence rates, conducted using regression models and random forests, revealed significant variations in vector-borne diseases incidence. An increase in dengue incidence (β = 213.24; 95% CI: 86.05-338.43), chikungunya (β = 26.41; 95% CI: 17.54-70.36), and Zika (β = 14.12; 95% CI: 10.06-89.30) was observed in years associated with ENSO events. In contrast, malaria (β = 17.28; 95% CI: 3.81-30.75), yellow fever, and cutaneous leishmaniasis (β = 8.67; 95% CI: 2.21-15.13) exhibited more heterogeneous patterns, influenced by variations in temperature, precipitation, and deforestation (figure 1).

### Association between El Niño and the incidence of diseases

**Figure 2:**
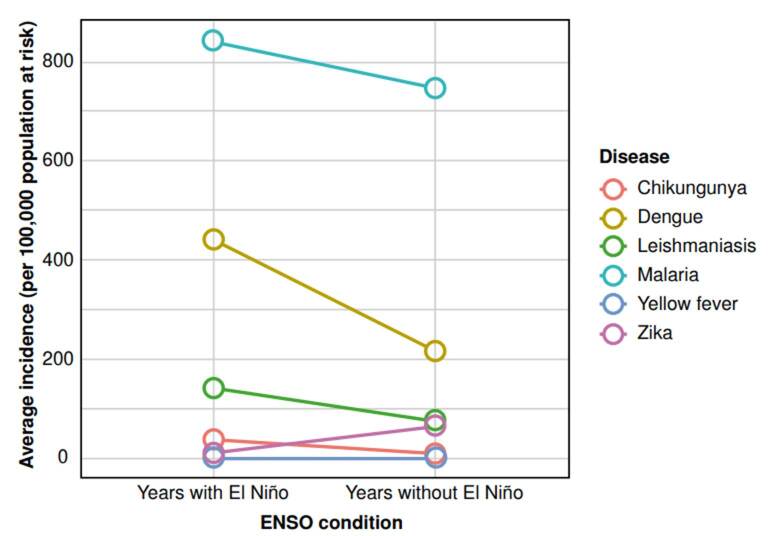
Comparison of the average incidence of each vector-borne disease in years with and without El Niño

The presence of ENSO was significantly associated with an increase in the incidence of dengue, chikungunya, and Zika (p < 0.05). However, in the case of malaria, yellow fever, and leishmaniasis, the relationship was less evident (table 2 and figure 2).

**Table 2:** Association between ENSO events and the incidence of vector-borne diseases in Colombia (2007-2024)

Disease	β coefficient	95% CI	p value
Dengue	213.24	86.05 - 338.43	< 0.05
Chikungunya	26.41	17.54 - 70.36	< 0.05
Zika	14.12	10.06 - 89.30	< 0.05
Leishmaniasis	74.36	-27.16 - 175.88	NS
Malaria	156.03	-43.38 - 355.44	NS
Yellow fever	0.0030	-0.0070 to - 0.0130	NS

NS: Not significant

Note: Coefficients derived from Poisson regression models adjusted for population size.

Statistically significant associations (p < 0.05) are highlighted in bold.

### Impact of environmental factors on the incidence of vector-borne diseases

Deforestation was a significant predictive factor for the incidence of leishmaniasis (β = 8.67; 95% CI: 2.21-15.13) and malaria (β = 12.35; 95% CI: 4.62-20.08), while its association with other diseases was inconclusive. Similarly, maximum temperature was positively associated with the incidence of dengue (β = 5.74; 95% CI: 2.15-13.63) and malaria (β = 17.28; 95% CI: 3.81-30.75). In contrast, mean precipitation had a more variable effect, showing negative associations with chikungunya (β = -3.25; 95% CI: -5.90 to -0.60) and leishmaniasis (β = -1.02; 95% CI: -2.91 to -0.87) (table 3).

**Table 3:** Multivariable associations between environmental predictors and the incidence of vector-borne diseases (2007-2024)

Disease	Deforestation β (95% CI)	Max temperature β (95% CI)	Mean precipitation β (95% CI)
Malaria	12.35 (4.62 - 20.08)*	17.28 (3.81 - 30.75)*	4.56 (1.32 - 7.80)*
Leishmaniasis	8.67 (2.21 - 15.13)*	-0.025 (-0.048 - 0.002)	-1.02 (-2.91 to -0.87)*
Dengue	-3.89 (-7.65 - 0.13)	5.74 (2.15 - 13.63)*	2.14 (-1.55 - 5.83)
Chikungunya	-5.21 (-9.75 - 0.67)	-10.16 (-18.25 - 2.07)	-3.25 (-5.90 to -0.60)*
Zika	-7.48 (-14.92 - 0.04)	-15.51 (-29.38 - 1.64)	-2.89 (-6.04 - 0.26)
Yellow fever	0.0003 (-0.0009 - 0.0015)	0.0002 (-0.0012 - 0.0016)	0.0001 (-0.0005 - 0.0007)

* Coefficients derived from Poisson regression models.

Statistically significant values (p < 0.05) are marked with an asterisk.

### Frequency of epidemic outbreaks and their relationship with El Niño

**Figure 3:**
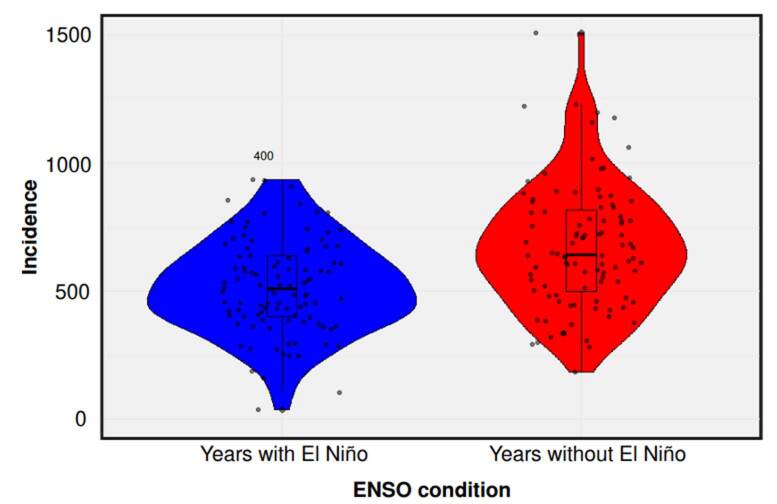
Incidence rates in years with and without El Niño

During the study period, 26 epidemic outbreaks were recorded, of which 30.8% were associated with dengue, 19.2% with malaria, and 11.5% with chikungunya. These outbreaks were more frequent in years affected by El Niño, indicating a potential link between anomalous climatic conditions and epidemic intensity (figure 3).

### Key factors in outbreak prediction

**Figure 4:**
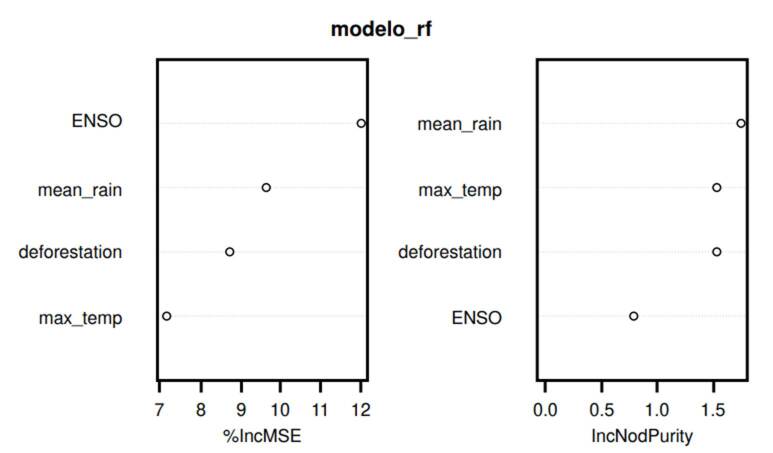
Variable importance in outbreak prediction (random forest)

The random forest model indicated that ENSO was the most influential variable in predicting outbreaks, followed by mean precipitation and deforestation (table 4 and figure 4).

**Table 4:** Multivariable regression model of environmental predictors associated with vector-borne diseases outbreak occurrence (2007-2024)

Variable	β estimate	95% CI	p value
Intercept	-9.322	-9.356 to -9.288	-
El Niño	0.4064	0.4029 - 0.4099	< 0.05
Deforestation	0.0001	0.0001073 - 0.0001097	< 0.05
Max temperature	0.0516	0.0505 - 0.0527	< 0.05
Mean precipitation	0.0194	0.0188 - 0.0199	< 0.05

Note: All predictors shown were statistically significant in the final model. Values were rounded to four decimals for interpretability.

## Discussion

This study analyzed the incidence and outbreaks of vector-borne diseases in Colombia between 2007 and 2024, exploring their relationship with climatic and environmental factors, with a particular focus on the impact of the ENSO phenomenon and deforestation ^15^. The findings indicate that increased temperature and reduced precipitation during ENSO years were strongly associated with a rise in the incidence of diseases such as dengue, chikungunya, and Zika. Additionally, deforestation emerged as a key factor, primarily affecting malaria and cutaneous leishmaniasis ^15^^-^^17^.

These results align with previous studies that have identified ENSO as a driver of dengue epidemics in Latin America, due to temperature and humidity conditions favorable for the proliferation of the *Aedes aegypti* mosquito ^18^^-^^20^. In the case of chikungunya and Zika, although with relatively fewer longitudinal studies, recent research has suggested a pattern similar to dengue in tropical regions ^21^^,^^22^. In this aspect, rising temperatures accelerate viral development within the mosquito and shorten the vector’s reproductive cycle, while reduced precipitation can lead to water accumulation in artificial containers, creating breeding sites for urban mosquitoes ^23^^,^^24^. This would explain why outbreaks and incidence of these arboviral diseases are more frequent during these periods.

It is important to highlight that chikungunya and Zika are relatively new diseases in Colombia, with the first autochthonous cases reported in 2014 and 2015, respectively ^25^; their introduction was accompanied by significant epidemic outbreaks due to the lack of population immunity and the widespread presence of *A. aegypti*^25^^,^^26^. While our analyses indicate that these viruses follow a pattern similar to dengue in relation to ENSO, the recent emergence of these diseases suggests that long-term trends should still be interpreted with caution. As population immunity accumulates and climatic conditions continue to change, the transmission dynamics may differ from those observed in this first decade after their introduction.

In contrast, malaria showed a less evident relationship with the ENSO phenomenon. While previous studies have suggested that changes in temperature and precipitation can influence *Plasmodium* spp. transmission ^27^, our results indicate that its incidence and outbreaks are more closely related to deforestation and alterations in the hydrography of endemic regions ^28^^,^^29^. This suggests that the expansion of the agricultural frontier and urbanization may be intensifying transmission.

The link between deforestation and malaria has been widely documented in the Amazon and other tropical regions, where the loss of vegetation cover creates more suitable habitats for *Anopheles* spp. vectors, altering their behavior and facilitating human contact ^30^^-^^32^. Additionally, in deforested areas, the presence of exposed water bodies with higher temperatures may favor vector larval development, suggesting that environmental transformation plays a more determining role than large-scale climate fluctuations ^30^^-^^32^.

Similarly, cutaneous leishmaniasis showed a stronger association with deforestation than with the ENSO phenomenon, supporting the hypothesis that ecosystem disruption increases human exposure to *Leishmania* spp. vectors (*Phlebotomus* spp. sandflies) ^6^^,^^33^. In Colombia, cutaneous leishmaniasis is endemic in various rural regions, and its transmission depends on the ecological balance between vectors, reservoirs, and humans.

The link with deforestation can be explained through several mechanisms. First, habitat fragmentation forces sandflies to adapt to more open and human-modified environments, increasing the likelihood of human contact ^6^. Second, human activities associated with deforestation, such as agricultural expansion, livestock farming, illegal mining, and infrastructure development, can increase local populations’ exposure to these vectors ^34^. Additionally, biodiversity loss may reduce the availability of natural reservoirs for the parasite, facilitating transmission to humans ^35^.

These findings suggest that, unlike arboviruses, where interannual climate variability plays a key role, cutaneous leishmaniasis is more closely linked to long-term environmental changes ^36^. This reinforces the need for public health strategies that not only respond to climatic factors but also integrate environmental conservation policies and vector control measures in deforested areas.

Another key finding is the importance of temperature as a predictor of incidence, particularly for dengue and malaria. Studies have shown that rising temperatures accelerate mosquito development and shorten the extrinsic incubation period of viruses and parasites within their vectors ^37^. For dengue, chikungunya, and Zika, this effect is amplified during ENSO years due to reduced precipitation and the proliferation of breeding sites in urban environments ^1^^,^^38^. For malaria, although no clear relationship with ENSO was observed, temperature remains a crucial factor, as it influences the development rate of the *Plasmodium* spp. parasite within Anopheles spp. mosquitoes and transmission dynamics ^39^. However, other elements may modulate its impact, such as ecosystem alterations due to deforestation, insecticide resistance, and human migration patterns ^40^. These results suggest the need for a more detailed approach to malaria studies, incorporating data on land-use changes, urbanization, and vector resistance to control interventions.

Unlike other arboviruses, yellow fever showed a less evident relationship with the analyzed climatic variables. This may be due to the complexity of its transmission, which involves both sylvatic cycles (with non-human primates as reservoirs and *Haemagogus* spp. and *Sabethes* spp. mosquitoes as vectors) and urban cycles (*A. aegypti* as the primary vector). Some studies have found that yellow fever outbreaks in South America may be influenced by changes in precipitation and temperature patterns ^41^^,^^42^, but in this analysis, no strong association with ENSO was identified. However, deforestation and habitat fragmentation have been documented to increase human exposure to sylvatic vectors ^43^, suggesting that additional ecological factors may be modulating the incidence of the disease in Colombia.

Another aspect to consider is the influence of yellow fever vaccination on epidemiological dynamics. Unlike diseases such as dengue and malaria, which lack widely implemented vaccines, yellow fever has an effective vaccine that may be reducing incidence in certain areas ^44^. However, in regions with insufficient vaccine coverage, outbreaks may be more frequent and more closely related to agricultural frontier expansion and human mobility.

This study presents some limitations that should be considered when interpreting the results. First, incidence data may be subject to underreporting or variability in epidemiological surveillance over time, which could affect the accuracy of the estimates. Second, although key climatic factors were included, other determinants such as human mobility, public health interventions, and socioeconomic dynamics were not analyzed in depth, which could influence the distribution and persistence of these diseases. Finally, the spatial scale of the analysis, focusing on departmental levels, might obscure relevant local patterns that require more detailed studies at the municipal or village level.

Nonetheless, this study has significant strengths. The distinction between outbreaks and incidence rates allowed for a better understanding of the transmission dynamics of vector-borne diseases. While outbreaks reflect a sudden increase in the number of cases over a short period, annual incidence captures the overall disease burden in the population, providing a comprehensive view of epidemiological behavior. This approach enhances the interpretation of associated factors and facilitates the design of differentiated strategies for the prevention and control of these diseases in various epidemiological and environmental contexts.

In conclusion, these findings underscore the need to integrate public health strategies with environmental conservation and climate change mitigation policies. Strengthening epidemiological surveillance during ENSO years is essential to anticipate vector-borne diseases outbreaks, while targeted vector control actions are especially needed in deforested areas where malaria and cutaneous leishmaniasis remain highly prevalent.

Given the context of climate change and accelerated environmental degradation, it is critical to develop localized predictive models that incorporate environmental, social, and health system variables. Moreover, deforestation should not be viewed solely as an ecological phenomenon but as a complex socio-environmental process driven by land use practices, economic activities, population dynamics, and governance factors, which interact with climate variability to amplify disease transmission risks.

From a policy perspective, the study results highlight the need to incorporate climatic and ecological indicators, such as ENSO alerts, temperature anomalies, and forest-cover loss, into national epidemiological surveillance system coordinated by the *Instituto Nacional de Salud*. Vector control actions should be aligned with the Decennial Public Health Plan (2022-2031), and vector-borne diseases risk mapping should inform the national zero deforestation strategy and territorial climate change plans. Furthermore, land-use planning instruments (e.g., *Plan de ordenamiento territorial*, and *Plan de ordenación y manejo de cuencas hidrográficas*) must embed epidemiological risk assessments to curb disease emergence in rapidly transforming territories.

Sustained, coordinated action among the health, environment, and planning sectors is essential to leverage shared data systems, maintain updated risk maps, and jointly monitor vector-borne diseases trends amid ongoing climatic and ecological pressures.
